# Where Do the Cultural Differences in Dynamics of Controlling Parenting Lie? Adolescents as Active Agents in the Perception of and Coping with Parental Behavior

**DOI:** 10.5334/pb.306

**Published:** 2016-07-13

**Authors:** Beiwen Chen, Bart Soenens, Maarten Vansteenkiste, Stijn Van Petegem, Wim Beyers

**Affiliations:** 1 Ghent University, BE; 2 University of Lausanne, CH

**Keywords:** Parenting, Control, Autonomy-Support, Psychological Control, Guilt-induction, Psychological needs, Coping, Cross-cultural, Self-Determination Theory

## Abstract

There is ongoing debate about the universal or culture-specific role of controlling parenting in children’s and adolescents’ development. This study addressed the possibility of cultural variability in how controlling parenting practices are perceived and dealt with. Specifically, we examined Belgian (*N* = 341) and Chinese (*N* = 316) adolescents’ perceptions of and reactions towards a vignette depicting parental guilt-induction, relative to generally controlling and autonomy supportive vignettes. Whereas Belgian adolescents perceived guilt-induction to be as controlling as generally controlling parental behavior, Chinese adolescents’ perception of guilt-induction as controlling was more moderate. Belgian and Chinese adolescents also showed some similarities and differences in their responses to the feelings of need frustration following from the controlling practices, with compulsive compliance for instance being more common in Chinese adolescents. Discussion focuses on cross-cultural similarities and differences in dynamics of controlling parenting.

Research increasingly suggests that, when children perceive their parents as controlling (i.e., as pressuring, intrusive, and domineering), they are more likely to display maladjustment, an effect that has been observed across countries with a different cultural climate (e.g., Pomerantz & Wang, 2009). To explain these findings, it has been argued that controlling parenting may frustrate universal psychological needs in children, that is, the needs for autonomy, competence, and relatedness (e.g., Deci & Ryan, 2000; Soenens & Vansteenkiste, 2010). However, these universal effects of perceived controlling parenting do not preclude the possibility of cross–cultural differences (a) in the way adolescents come to perceive parental behavior as controlling and (b) in the way they respond to controlling parenting (Soenens, Vansteenkiste, & Van Petegem, 2015). This study aimed to tap into these more specific and possibly culture-dependent processes. We explored these processes with a focus on parental guilt-induction, a parenting practice that is assumed to be characteristic of controlling parenting (e.g., Barber, 1996) and that may operate differently in different cultures (e.g., Fung & Lau, 2012). Specifically, we compared Chinese and Western-European (i.c., Belgian) adolescents’ perceptions and ways of coping in response to perceived guilt-induction, relative to more general forms of controlling parenting and relative to parental autonomy support (i.e., parenting that supports children’s volitional functioning).

## Controlling Parenting and Adolescents’ Psychosocial Adjustment

To unravel some of the complexities of the concept of parental control (e.g., Barber, 1996; Grolnick & Pomerantz, 2009; Soenens & Vansteenkiste, 2010), we rely on Self-Determination Theory (SDT; Deci & Ryan, 2000), a general theory of motivation that is increasingly applied to the domain of socialization and parenting. In SDT, controlling parenting is characteristic of parents who pressure their children to think, behave, or feel in particular ways (Grolnick, Deci, & Ryan, 1997). Controlling parenting is contrasted with autonomy-supportive parenting, which is characteristic of parents who promote volitional functioning and self-endorsement in their children by encouraging initiative, providing choices, acknowledging the child’s feelings and ideas, and providing a meaningful rationale when introducing a request (Grolnick et al., 1997; Soenens et al., 2007).

According to SDT, controlling parenting can manifest in a variety of ways, including (a) relatively more externally controlling and overtly punitive discipline techniques (such as corporal punishment or verbal hostility) and (b) relatively more internally controlling and insidious tactics such as shaming and love withdrawal (Grolnick, 2003; Soenens & Vansteenkiste, 2010). Using this SDT-based definition of controlling parenting, the concept of parental psychological control (Barber, 1996; Barber & Harmon, 2002) can be regarded as an important manifestation of controlling parenting. Psychological control is defined as “parental behaviors that are intrusive and manipulative of children’s thoughts, feelings, and attachments to parents” and involves components such as love withdrawal, shaming, excessive pressure for change, invalidation of feelings, and guilt-induction (Barber & Harmon, 2002, p. 15).

Accumulating evidence, part of which is based on research on parental psychological control and much of which is based on Western samples, has shown that perceived controlling parenting relates to internalizing problems such as depression, low self-esteem and anxiety (e.g., Barber, Stolz, & Olsen, 2005) as well as to externalizing problems, including delinquency, antisocial behavior, and substance use (e.g., Walker-Barnes & Mason, 2004). In contrast, perceived autonomy-supportive parenting has been found to relate to adaptive motivational and developmental outcomes in a variety of life domains (e.g., Grolnick, 2003).

Although controlling parenting occurs more frequently in Eastern compared to Western societies (e.g., Ng, Pomerantz, & Deng, 2013), the negative effects of perceived controlling parenting were found to generalize to relatively collectivistic societies (e.g., Ahmad, Vansteenkiste & Soenens, 2013; Barber, Stolz, & Olsen, 2005; Soenens, Park, Vansteenkiste, & Mouratidis, 2012). For instance, in both samples from the US and China, parental psychological control predicted decreases in children’s academic and emotional adjustment (Wang, Pomerantz, & Chen, 2007). In contrast, research increasingly shows that perceived autonomy-supportive parenting is related to beneficial outcomes across countries and cultures (Pomerantz & Wang, 2009).

## Psychological Need Frustration as a Mechanism Underlying Effects of Controlling Parenting

According to SDT, the underlying mechanism of the seemingly universal effect of controlling parenting is frustration of children’s psychological needs (Ryan, Deci, & Vansteenkiste, 2016; Soenens & Vansteenkiste, 2010). SDT maintains that human beings have a set of basic and innate psychological needs, the satisfaction of which represents a universally essential condition for well-being. In contrast, frustration of these basic needs is said to relate to defensiveness and ill-being (Ryan & Deci, 2000; Vansteenkiste & Ryan, 2013). First, the need for relatedness refers to experiences of intimacy and genuine connection with others. Frustration of the need for relatedness involves experiences of relational tension and loneliness. Second, the need for competence involves feeling effective and capable to achieve desired outcomes, whereas competence frustration involves feelings of failure and inefficacy. Third, the need for autonomy refers to experiences of self-endorsement and authenticity when carrying out an activity. Autonomy frustration, on the other hand, involves feeling coerced through externally enforced or self-imposed pressures. Across various cultures and countries, including non-Western countries, there is robust evidence that satisfaction of these needs is related to more adaptive motivational regulation of behavior (e.g., Delrue et al., 2016; Michou, Matos, Gargurevich, Gumus, & Herrera, 2016, this issue; Ryan & Deci, 2000) and to well-being and adjustment (e.g., Chen et al., 2015; Chirkov, Ryan, & Sheldon, 2010; Cordeiro, Paixao, Lens, Lacante, & Luyckx, 2016, this issue).

The identification of three universally essential needs may allow for an understanding of why perceived controlling parenting relates to maladjustment across cultures (Soenens & Vansteenkiste, 2010). When parents are perceived as controlling (i.e., pressuring), children feel compelled to do things that they have little interest in or do not value (i.e., autonomy need frustration). Controlling parenting often involves a conditional orientation towards the child, where the child feels that the parent only cares about him or her when meeting parental standards for conduct (Assor, Roth, & Deci, 2004). As such, it would lead to feelings of alienation from the parent and frustrate the need for relatedness. Controlling parenting can also frustrate children’s competence, in particular when their parents show disappointment and induce shame. In contrast to controlling parenting, autonomy-supportive parenting would contribute to satisfaction of the three needs (Grolnick et al., 1997). Consistent with this reasoning, a number of studies have shown that autonomy-supportive parenting predicted psychological need satisfaction which, in turn, relates to adaptive outcomes (e.g., Soenens et al., 2007). Controlling parenting, in contrast, has been found to relate to lower need satisfaction (or even need frustration) and subsequent maladjustment (e.g., Ahmad et al., 2013).

## Cultural Differences in the Perception of Controlling Parenting

In spite of the evidence for the presumably universal effects of perceived controlling parenting, some cross-cultural studies also found that the effect of controlling parenting was less negative or even absent in Eastern Asian samples (e.g., Chao, 1994; Iyengar & Lepper, 1999). One interpretation of these findings is that certain components of controlling parenting have a relatively less pressuring and more benign meaning in East Asian cultures (Grusec, 2012; Mason et al., 2004; Park & Kim, 2004). Chao (1994), for instance, proposed that Asian children interpret potentially controlling parenting more as parental concern or involvement. In contrast, in an individualistic culture such parenting would have a less benign meaning and would be more likely to be perceived as a reflection of parental anger and rejection. To test this interpretation, studies have begun to investigate cultural differences in the affective interpretation of potentially controlling parental behaviors (e.g., Camras, Sun, Li, & Wright, 2012; Chao & Aque, 2009; Helwig, To, Wang, Liu, and Yang, 2014; Mason et al., 2004). For instance, Helwig, To, Wang, Liu, and Yang (2014) found that rural Chinese adolescents had a more favorable evaluation of the practice of love withdrawal (i.e., an element of psychologically controlling parenting) than urban Chinese and Canadian adolescents.

Along similar lines, SDT highlights the difference between the occurrence of an event and its *functional significance* (Deci & Ryan, 1987). Contextual events such as parental behaviors can be experienced in different ways because the behaviors come to have different meanings in accordance with culturally endorsed values (Deci & Ryan, 1987; Ryan & Deci, 2006). The notion of functional significance has important repercussions for the debate about whether the effect of controlling parenting is culture-bounded or universal (Soenens et al., 2015). Whereas *perceived* controlling parenting might represent a culturally invariant antecedent of maladjustment, there is room for cultural variation in how people *subjectively* experience potentially controlling *objective* parental behaviors. In other words, by separating objective parental behaviors from their subjective perception, we may be able to reconcile culture-specific and universal-process perspectives (Soenens et al., 2015). In this study, we aimed to examine cross-cultural differences in Chinese and Belgian adolescents’ perception of the practice of guilt-induction. We focused on guilt-induction because this parental strategy in particular might be interpreted in various ways and, as a consequence, might be especially prone to cross-cultural variability (Fung & Lau, 2012).

## Guilt-Induction as an Ambivalent Facet of Controlling Parenting

Guilt-induction, a parenting practice often mentioned as part of the construct of parental psychological control, refers to parental use of guilt as a means of pressuring children to comply with parental requests (Barber & Harmon, 2002). Compared to more explicit and overt forms of controlling parenting, such as the use of forceful language (e.g., “you should”) or the use of threats or punitive discipline (e.g., “you have to, otherwise …”), guilt-induction may be more of a mixed blessing (Nelson, Yang, Coyne, Olsen, & Hart, 2013). On the one hand, it may be perceived as having informational value because it conveys parents’ expectations of interpersonal obligation (Fung & Lau, 2012). On the other hand, guilt-induction may also have a connotation of parental controllingness and negative parental evaluation because parental love and appreciation are withdrawn (and replaced with parental disappointment) when children fail to comply with parental expectations (Baumeister, Stillwell & Heatherton, 1994).

Clearly, there is room for individual interpretation of the practice of guilt-induction and some of this variation in interpretation may depend on cultural background. Some scholars argue that especially in Asian societies, the feeling of guilt is a moral emotion which is indicative of filial piety and loyalty vis-à-vis the parents and that guilt may be useful to meet expectations about interpersonal obligation (Azuma, 1988; Park & Kim, 2004; Kim, Yang, & Hwang, 2006; Miller, Chakravarthy, & Das, 2008). Interpersonal obligation is considered to be a culturally desirable value that fits with a cultural emphasis on interdependence and relational closeness, especially within the family (Markus & Kitayama, 1991; Miller et al., 2008). Thus, adolescents in collectivist cultures may be less inclined to perceive parental guilt-induction negatively or as strongly controlling. Consistent with this reasoning, Rudy and Halgunseth (2005) reported that guilt-induction not only happens more frequently in collectivistic (i.e., India and Pakistan), relative to individualistic (i.e., Canadian and British), cultures but also that the use of guilt-induction was unrelated to maladaptive maternal cognitions. Furthermore, guilt-induction was found to be associated less strongly with harsh psychological control in Indian adolescents than in US adolescents (Rudy, Carlo, Lambert, & Awong, 2014). As Miller et al. (2008, p. 236) put it, “…there is a greater tendency for individuals to associate duty and guilt with satisfaction in the context of being responsive to the needs of family and friends.” These differential associations of guilt-induction may be due to the different ways in which guilt-induction is perceived by adolescents in different cultures. In the present study, we aim to examine directly whether East Asian (i.e., Chinese) adolescents perceive parental guilt-induction as less controlling than do Western (i.e., Belgian) adolescents.

In spite of the anticipated cultural differences in how guilt-induction is perceived, we hypothesized that perceived parental controllingness would relate to psychological need frustration in both Belgian and Chinese adolescents. On the basis of SDT, it can indeed be predicted that, as soon as parental behavior is experienced subjectively as controlling, need frustration is likely to occur, an effect that is expected to be universal (Deci & Ryan, 1987; Soenens et al., 2015).

## Coping with Psychological Need Frustration

In addition to examining cross-cultural differences in perceptions of controlling parenting, we examined cross-cultural differences in the way adolescents cope with the experience of need frustration, an issue that has received little empirical attention. One relevant framework in this regard is Skinner and colleagues’ coping theory (e.g., Skinner & Zimmer-Gembeck, 2007). Consistent with SDT, objective events are said to be stressful and threatening to the extent that the basic psychological needs get thwarted which, in turn, may trigger specific coping reactions (Skinner & Edge, 2002). Need frustration may trigger either oppositional defiance, which refers to doing exactly the opposite of what the situation demands (e.g., Ryan et al., 2016; Vansteenkiste & Ryan, 2013), or compulsive compliance, which involves a rigid obedience to the demand, thereby giving up one’s personal preferences (e.g., Grusec & Kuczynski, 1997). Yet, a more adaptive coping strategy involves negotiation, which means constructively articulating one’s own interests in order to reach a consensus (Skinner & Edge, 2002). The specific coping reaction that gets triggered would be function of a number of personal and contextual factors, including cultural background (Skinner & Zimmer-Gembeck, 2007).

In the cross-cultural literature, East Asian children are depicted as dutiful and obedient, an orientation that is influenced by the Confucian philosophy and family culture (e.g., Li, 2005). According to this view Asian adolescents are socialized to respect and follow parental guidance to demonstrate their filial piety (Wang & Hsueh, 2000). As a consequence, Chinese adolescents might be more likely to display unquestioning obedience, which would manifest as compulsive compliance when reacting to experienced need frustration vis-à-vis the parent.

In contrast, Chinese adolescents might be relatively less likely to respond to need frustration with oppositional defiance. Past work among Western adolescents found controlling parenting to elicit oppositional defiance through need frustration (Van Petegem, Soenens, Vansteenkiste, & Beyers, 2015). However, such a response may not necessarily apply to East Asian adolescents, as they are more oriented towards compliance to parents. Consistent with this reasoning, Pomerantz and Qin (2011) found that Chinese adolescents’ feelings of obligation to their parents increased with age, whereas they decreased with age in American adolescents. Extrapolating from this work, we tested in the current study whether Chinese, relative to Belgian, adolescents would less easily display oppositional defiance subsequent to need frustration.

Negotiation is said to represent a more constructive coping strategy (Compas, Connor-Smith, Saltzman, Thomsen, & Wadsworth, 2001). Negotiation involves expressing one’s personal perspective and wishes through a respectful dialogue (Skinner & Edge, 2002), a skill that is more highly valued in horizontal individualistic cultures (Oyserman et al., 2002). In light of this reasoning, Belgian, relative to Chinese adolescents, may engage in more negotiation in response to controlling parenting. On the other hand, Helwig et al. (2003) found that Chinese children do value concepts of rights, individual autonomy, and democracy in their social reasoning and also use these values to evaluate social practices. Accordingly, Chinese adolescents may also use negotiation as a way to maintain personal jurisdiction. Given these conflicting perspectives, the question whether there are cross-cultural differences in the use of negotiation was examined in a more explorative fashion.

## The Present Study

To better understand the functional role of guilt-induction across cultural contexts, it is important to separate the objective presence of guilt-induction from the subjective perception of this practice (Soenens et al., 2015). Whereas associations between the objective presence of parental guilt-induction and the perception of parental controllingness might be different across cultures, the link between perceived controlling parenting and psychological need frustration could be relatively invariant across cultures. In addition, there could be cultural variations in how adolescents cope with need frustration following perceived controlling parenting. An examination of these issues can contribute to a more nuanced view on cultural differences in parenting because attention is paid to both universal and culture-specific processes.

**Figure 1:**
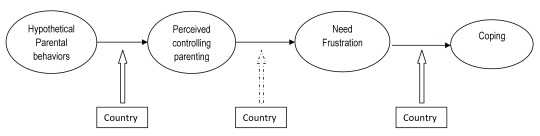
Hypothesized Integrated Model.

To examine these questions, we presented adolescents from China and Belgium with vignettes. An advantage of this approach is that it allows one to disentangle what parents actually say and how it is perceived and dealt with (Kakihara & Tilton-Weaver, 2009). Specifically we presented a vignette depicting either parental guilt-induction, or a more generally controlling vignette, or an autonomy-supportive vignette. In the vignettes we focused on the academic domain for two reasons. First, we anticipated that there would be room for cross-cultural differences in this domain as academic success is associated with a greater sense of morality and responsibility in China compared to Western countries (e.g., Pomerantz & Wang, 2009). Second, across cultures academic functioning is an important domain in adolescents’ lives and a domain in which parents are often involved (e.g., Pomerantz, Moorman, & Litwack, 2007). After reading the vignettes, participants rated the perceived controllingness of the parental behavior, need frustration vis-à-vis the parent, and engagement in different coping responses. The basic model integrating all of the study variables to be examined is depicted in Figure 1. Specifically, we examined the two sets of hypotheses and one more explorative research question.

First, we examined whether Belgian and Chinese adolescents would perceive the guilt-induction vignette differently in terms of its controlling nature and experienced need frustration, as compared to the two other vignettes. We hypothesized that Belgian adolescents would experience guilt-induction as equally controlling and need frustrating as the generally controlling vignette, which both would differ from the autonomy-supportive vignette. Chinese adolescents, on the other hand, would interpret the guilt-induction vignette in a relatively more benign fashion. For Chinese adolescents, experiences associated with guilt-induction might take an intermediate position in between the autonomy supportive vignette and the more generally controlling vignette (Hypothesis 1). Second, we examined the relation between perceived controlling parenting and experiences of need frustration. Based on SDT and previous research, we hypothesized that perceived controlling parenting would relate to more need frustration and that this relation would be equivalent across the two samples (Hypothesis 2). Third, we examined potential cross-cultural similarities and differences in adolescents’ coping responses. We hypothesized that compulsive compliance would be a relatively more dominant coping response in Chinese adolescents. In other words, among Chinese participants the association of experiences of need frustration with compulsive compliance may be more pronounced than among Belgian participants. In contrast, we expected that oppositional defiance would be a relatively more dominant coping response in Belgian adolescents and that, accordingly, the association of oppositional defiance with need frustration would be stronger in the Belgian sample. As we did not have explicit hypotheses about the role of negotiation, we examined cross-cultural differences in negotiation in a more explorative fashion (Research Question 1).

## Method

### Participants

Participants were 341 Chinese adolescents and 316 Belgian adolescents. The Belgian sample was 40.5% male and the mean age of participants was 15.74 years (range = 12 to 19 years, *SD* = 1.16). In the Belgian sample, 78.9% of the participants came from intact families, 18% came from a divorced family, and 1.9% came from a family in which one parent had deceased. Almost all participants (96.8%) in the Belgian sample had the Belgian nationality. The Chinese sample was 50.2% male and the mean age of participants was 14.39 years (range = 14 to 16 years, *SD* = 0.51). In the Chinese sample, 79.4%, of the participants came from an intact family, 8.3% from a divorced family, and 1.4% from a family in which one parent had deceased. All participants in the Chinese sample were of Chinese nationality.

Of all the participants, only one showed missing data on more than 20% of the variables and was therefore not retained in the analyses. Little’s (1988) MCAR-test produced a normed χ^2^ (χ^2^/df) of 1.19 for the remaining participants. According to Bollen (1989), this indicates that the data were likely missing at random, and as a consequence, missing values could be estimated and cases with missing values could be retained in the analyses. To do so, we used the Full Information Maximum Likelihood (FIML) procedure available in Mplus 7 (Muthén & Muthén, 2007).

### Procedure

In Belgium, the data were gathered in the context of a course on developmental psychology. Specifically, participants lived in the Dutch-speaking part of Belgium (Flanders). Trained undergraduate students visited the families at home to administer the questionnaires. An informed consent form was signed by both the adolescents and the parents. Adolescent participants received a questionnaire package with the purpose of the study and brief instructions provided on the first page. In China, the data were gathered at a middle school with mainly middle-class students in an urban Chinese area (Shanghai). During a regular class period, an assistant researcher administered the questionnaires package with the purpose of the study and brief instructions on the first page, which were exactly the same as those in the Belgian sample. In both countries, the questionnaire package was designed to take about half an hour to complete. All participants were assured that participation was voluntary and that their data would be treated confidentially. Adolescents were randomly distributed to the three conditions. They received either a questionnaire package with an autonomy-supportive vignette (27.3% and 33.8% of the Belgian and Chinese samples, respectively), a generally controlling vignette (27.9% and 29.6%, respectively), or a guilt-inducting vignette (44.8% and 36.6%).

### Materials

**Vignettes.** Three vignettes were used to operationalize three different maternal styles of communication, that is, a generally controlling style, a guilt-inducing style, and an autonomy-supportive style. Common to the three vignettes was the description of a situation in which the child had obtained a lower grade than usual for an important course at school. Specifically, participants were asked to imagine the following situation: “*One day you come home from school with a lower grade than usual for an important course. Because initially you thought the test went fairly well, you expected good points, and this is also what you told your mother. When you now inform your mother about your low grade, here is what she says: […].”* Following the description of the situation, participants read a maternal request to study more on a next occasion. There were three different styles in which the request was formulated, that is, autonomy-supportive (e.g., inviting language, acknowledgement of other’s perspective), guilt-inducing (e.g., expressing disappointment and highlighting parental sacrifices made for the child in the past), or generally controlling(i.e., a mixture of expressing disappointment, lack of perspective taking, blaming, controlling language and intrusive monitoring). The specific responses are presented in Appendix 1.

Information about the development and pilot testing of the controlling and the autonomy-supportive vignettes is presented in Van Petegem et al. (2015). The vignette for guilt-induction was constructed for the purpose of the present study. To develop the guilt-induction vignette, we relied on conceptual descriptions of guilt-induction available in the literature (e.g., Barber et al., 2002) and we adopted a simultaneous approach, which means developing the different language versions at the same time by moving back and forth among three languages (i.e., English, Dutch, and Chinese) and between the Belgian and Chinese culture to minimize cultural bias (Harkness, Van de Vijver, & Johnson, 2002). Specifically, researchers from different cultural and language backgrounds discussed together which situation to choose and how to formulate the vignette to capture the main concepts equivalently in different languages and cultures.

The vignettes and questionnaires were originally developed in English and translated to Dutch for Belgian adolescents and to Chinese for Chinese adolescents. Back-translation procedures (Brislin, 1970) were adopted to ensure conceptual equivalence across languages. In both cases, the questionnaire was translated from English into Dutch or Chinese by a bilingual researcher and then translated back to English by another bilingual graduate student. The back-translated and original questionnaires were compared and points of discrepancy were discussed to reach consensus in accurate reflection of the original meaning in the English questionnaire.

**Perceived Controlling Parenting.** To assess adolescents’ perception of the parental situation as controlling, we used two items from the Psychological Control Scale – Youth Self-Report (PCS – YSR; Barber, 1996) and two items from the Perceptions of Parents Scales (POPS; Grolnick, Deci, & Ryan, 1997). These items seemed most directly relevant to the current study and have been used previously in a vignette-based study on the dynamics of controlling parenting (Van Petegem et al., 2015). Specifically, following an item stem ( “*If my mother reacts like this, I would feel like… . .”*), participants rated the following 4 items: *“she insists upon doing things her way*”, *“she is not very sensitive to my needs” “she is disappointed with me”, “she is trying to change how I see things”*. Cronbach’s alpha was for .84 the Belgian sample and .60 for the Chinese sample.

**Psychological Need Frustration.** To assess basic psychological need frustration, we used items from the Basic Psychological Need Satisfaction and Frustration Scale (BPNSNF) which has been validated across four countries (i.e., Peru, China, US, Belgium; Chen et al., 2015). The BPNSNF consists of three subscales pertaining to the frustration (versus satisfaction) of each of the three needs identified in SDT. For the purpose of this study, we used 9 items tapping into frustration of the basic psychological needs for autonomy^1^, relatedness and competence, and we reformulated the items such that they refer to the specific situation described in the vignettes. Items were selected on the basis of their relevance to the context of parent-child relationships and with the purpose of minimizing ambiguity due to cultural differences. All items again followed the item stem *“If my mother would react like this …”*). An example of autonomy need frustration is: *“I would feel forced to do things I wouldn’t choose to do*”. An example of relatedness need frustration is: *“I would feel excluded by my mother”*. An example of competence need frustration is: *“I would feel disappointed with my performance”*. We computed a total score for need frustration (a) because there were strong correlations between scores for frustration of the three needs and (b) because theoretically it is assumed that controlling parenting thwarts each of the three needs (Soenens & Vansteenkiste, 2010). Cronbach’s alpha was .87 in the Belgian sample and .82 in the Chinese sample.

**Coping.** Each of the three coping styles was assessed with 4 items. Oppositional defiance was measured with a scale developed and validated by Vansteenkiste et al. (2014). An example item is *“I would rebel against the request of my mother”*. Items for compulsive compliance and negotiation were inspired by Skinner and colleagues’ theoretical framework and by the Child Coping Questionnaire developed by Finnegan, Hodges, and Perry (1998). Example items are: *“I would do what she expects from me, even if what she says is not meaningful to me”* (compulsive compliance) and *“I would explain to my mother how I think about it”* (negotiation). Cronbach’s alpha for three coping styles was for .75, .82 and .86 respectively for the Belgian sample and .75, .85 and .88 for the Chinese sample.

## Results

### Veridicality of the Situation in the Vignettes

To check the veridicality of the hypothetical situation which formed the basis of all vignettes (an adolescent showing his/her poor exam results to parents), participants first evaluated this basic situation on two criteria, that is, relevance (*“How relevant is the situation as such in your life?”*) and credibility (*“Do you think youngsters at your age ever experienced such a situation?”*), thereby using a 7-point scale ranging from “*completely disagree*” to “*completely agree*”. The situation itself was rated as relatively relevant by both the Belgian (*M* = 5.05, *SD* = 1.30) and Chinese adolescents (*M* = 5.21, *SD* = 1.77) and both samples did not differ from each other, *F*(1,645) = 1.70, *ns*. Moreover, the situation was rated as believable by both the Belgian (*M* = 5.99, *SD* = 1.03) and Chinese adolescents (*M* = 5.82, *SD* = 1.54) and both samples did not differ from each other, *F*(1,647) = 2.58, *ns*. There were also no significant between-condition differences in relevance and credibility, which is logical because this basic situation was exactly the same in each of the three conditions.

In addition, participants rated how frequently each of the three parental responses occurred on a 5-point scale, ranging from 1 (*“never”*) to 5 (*“always”*). The following means were obtained among the Belgian adolescents, *M*_AS_ = 3.34 (*SD* = 1.22), *M*_Guilt_ = 2.59 (*SD* = 1.19) and *M*_CON_ = 2.68 (*SD* = 1.37), and Chinese adolescents, *M*_AS_ = 2.78 (*SD* = 1.26), *M*_Guilt_ = 2.70 (*SD* = 1.38) and *M*_CON_ = 2.60 (*SD* = 1.19). ANOVAs showed that the two samples differed significantly on the reported frequency of the autonomy-supportive response [*F*(1,175) = 7.50, *p* < .01, *η^²^* = .04], with the autonomy-supportive response being more common in Belgium. The frequency of the two controlling responses did not differ between the samples.

### Descriptive Statistics and Background Variables

Descriptive statistics and correlations between the study variables can be found in Table 1. We examined whether gender and age would be related to the study variables. A MANOVA with gender as an independent variable and all study variables as dependent variables revealed a multivariate effect, Wilk’s Lambda *F*(6,591) = 4.21, *p* < .01, η^2^ = .04. Subsequent univariate ANOVAs showed that boys (*M* = 2.17, *SD* = 0.92) reported more oppositional defiance than girls (*M* = 1.91, *SD* = 0.79), *F* (1,596) = 13.60, *p* < .05, η^2^ = .02. No other gender effects were found. As for age effects, correlations showed that age was correlated positively with perceived controlling parenting (*r* = .09, *p* < .01), and negotiation (*r* = .10, *p* < .01), while it was negatively associated with compulsive compliance (*r* = .15, *p* < .01). In the main analyses, we controlled for both gender and age.

**Table 1:** Descriptive Statistics and Correlations between The Study Variables Across Situations.

	*M (SD)* Belgium	*M (SD)* China	1	2	3	4	5

Perceived controlling parenting	3.00 (0.98)	2.91 (0.85)	1	.77***	–.12*	.39***	.06
Need frustration	2.76 (0.79)	2.74 (0.78)	.60***	1	–.04	.35***	.01
Compulsive compliance	2.65 (0.74)	2.85 (0.98)	.34***	.36***	1	–.35***	–.28***
Oppositional defiance	2.06 (0.79)	1.99 (0.95)	.25***	.36***	–.05	1	–.14**
Negotiation	3.84 (0.72)	3.52 (1.08)	–.36***	–.34***	–.10	–.30***	1

*Note.* Above diagonal are correlations in the Belgian data; below diagonal are correlations in the Chinese data. *p < .05, **p < .01, ***p < .001.

### Between-Vignette Differences in Perceptions and Coping Responses

To examine between-vignette differences in terms of perceived controlling parenting, need frustration, and coping, we first performed a MANCOVA with both type of vignette and country as independent fixed factors, while controlling for gender as an independent fixed factor and age as a covariate. The adjusted mean scores for the outcomes per vignette and per country are presented in Table 2. First, we examined the main effect of vignette type on the outcomes in the total sample. The MANCOVA indicated a significant multivariate effect of type of vignette, Wilk’s Lambda *F*(12,1154) = 5.50, *p* < .01, η^2^ = .05. There was no significant interaction between type of vignette and gender, Wilk’s Lambda *F*(12,1154) = 1.44, *ns*, nor between type of vignette and age, Wilk’s Lambda *F*(18,1632) = 0.98, *ns*. Although these interactions with gender and age were non-significant, gender and age might still be confounding variables and we therefore controlled for gender and age in all further analyses.

**Table 2:** Main Effects of Situation and Interaction with Country (MANCOVA).

Dependent variables		*M (SD)*			Between-vignette differences	Country x Vignette effects
			
		Autonomy-support	Guilt-induction	Generally controlling	*F*(2,*n*−2)	*F*(2,652)

Perception of the situation						
Controlling parenting	Total	2.32 (0.70)^a^	3.13 (0.76)^b^	3.31 (0.70)^b^	83.06***	12.12***
	Belgium	2.09 (0.86)^a^	3.30 (0.85)^b^	3.34 (0.82)^b^	83.58***
	China	2.55 (0.97)^a^	2.96 (0.92)^b^	3.29 (0.85)^c^	16.54***
Need frustration	Total	2.32 (0.86)^a^	2.89 (0.82)^b^	3.00 (0.80)^b^	55.65***	15.90***
	Belgium	2.04 (0.51)^a^	3.01 (0.72)^b^	2.91 (0.70)^b^	68.59***
	China	2.60 (0.80)^a^	2.77 (0.76)^a^	3.09 (0.70)^b^	12.11***
Coping responses									
Compulsive compliance	Total	2.63 (0.83)^a^	2.80 (0.84)^ab^	2.84 (0.92)^b^	4.31*	6.76**
	Belgium	2.75 (0.69)^a^	2.71 (0.73)^a^	2.64 (0.80)^a^	0.35
	China	2.51 (0.95)^a^	2.89 (0.94)^b^	3.05 (0.98)^b^	9.75***
Oppositional defiance	Total	1.89 (0.82)^a^	2.08 (0.82)^ab^	2.13 (0.93)^b^	4.47*	0.79
	Belgium	1.85 (0.72)^a^	2.07 (0.73)^ab^	2.19 (0.92)^b^	4.48*
	China	1.93 (0.90)	2.09 (0.94)	2.06 (0.95)	0.70
Negotiation	Total	3.66 (0.95)^a^	3.63 (0.87)^a^	3.70 (0.94)^a^	0.07	2.06
	Belgium	3.71 (0.71)	3.82 (0.71)	3.94 (0.74)	2.21
	China	3.61 (1.14)	3.45 (1.02)	3.47 (1.07)	0.65

*Note.* Means within rows with different superscripts are significantly different (post hoc Tukey contrasts; *p* < 05). *p < .05, **p < .01, ***p < .001.

More importantly and as expected, the MANCOVA indicated a significant multivariate interaction between type of vignette and country, Wilk’s Lambda *F*(6,614) = 6.55, *p* < .01, η^2^ = .06, indicating that at least some of the between-vignette differences were qualified by country. Subsequent univariate ANOVAs showed that the interaction effects were significant for perceived controlling parenting, need frustration, and compulsive compliance. As shown in Table 2, Belgian adolescents perceived the generally controlling and guilt-inducing vignette to be more controlling and to yield more need frustration compared to the autonomy supportive vignette, whereas there were no significant differences between the generally controlling vignette and the guilt-inducing vignette. Said differently, Belgian adolescents perceived and experienced guilt-induction to be similar to a generally controlling style, thus supporting Hypothesis 1. In contrast, Chinese adolescents perceived the generally controlling vignette to be more controlling compared to the guilt-inducing vignette which, in turn, differed from the autonomy-supportive vignette. In terms of perceived need frustration, Chinese participants reported more need frustration in the generally controlling vignette compared to the guilt-induction vignette, which did not differ from the autonomy-supportive vignette. Overall then, Chinese adolescents perceived guilt-induction as more favorable than the generally controlling vignette and as less favorable than the autonomy-supportive vignette.

As for the coping responses, Belgian adolescents’ report of compulsive compliance did not differ between the three situations, whereas Chinese adolescents reported more compulsive compliance in response to the guilt-induction and generally controlling vignettes compared to the autonomy-supportive vignette. The between-vignette differences in oppositional defiance were not moderated by country. Across the two countries, adolescents reported more defiance in the generally controlling than in the autonomy-supportive vignette. Finally, neither the type of vignette nor the interaction between vignette and country affected scores on negotiation.

### Structural Equation Models (SEM)

We aimed to test the proposed integrated model (Figure 1) with multi-group SEM analysis (with country as a moderator). Accordingly, we used the Mplus 7.4 software with robust maximum likelihood estimation, which can correct for the observed non-normality of the variables (Muthén & Muthén, 2007). Prior to testing the structural models, we first tested for metric equivalence of the measurement model by constraining the factor loadings of the items to each latent construct to be equal across the two groups. When metric invariance is reached, it is legitimate to compare the relations between latent variables across groups. We compared a constrained model to a model without constraints. We took the difference in the Satorra-Bentler scaled chi-square statistic (ΔSBS-χ^2^) as the criterion for model comparisons. The constrained measurement model had an acceptable fit, SBS-χ^2^ (532) = 1028.39, CFI = .915, RMSEA = .05, SRMR = .07. However, compared to the fit of the nested unconstrained model, SBS-χ^2^ (512) = 965.31, CFI = .923, RMSEA = .05, SRMR = .06, the constrained model had a significantly worse fit, ΔSBS-χ^2^ (20) = 63.08, *p* < .01. Modification indices suggested that the difference between both models was in particular due to one item from the scale for perceived controlling parenting (“*She is trying to change how I see things”*), which had different loadings across the groups. As a result, we removed this non-equivalent item in the main analyses.

**Figure 2:**
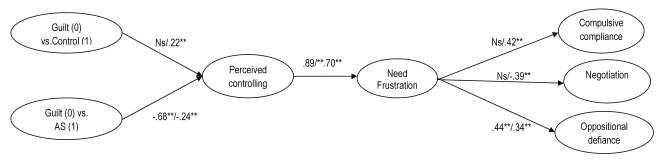
SEM Model. *Note.* *p < .05; **p < .01, ***p < .001. The first coefficient refers to the Belgian sample and the second coefficient refers to the Chinese sample. SBS-χ^2^ (403) = 719.54, *p* < .01; CFI = .93; SRMR = .07; RMSEA = .05; AS = Autonomy-Support.

Next, we tested the structural models, controlling for age and gender. To represent the three levels of the type of vignette variable, we took the guilt-induction vignette as the main reference point and we created two dummies, one comparing guilt-induction (0) to the generally controlling vignette (1) and one comparing guilt-induction (0) to the autonomy-supportive vignette (1). First, we examined the model in the total sample. The model fitted the data well, with SBS-χ^2^ (194) = 476.23, *p* < .01; CFI = .93; SRMR = .05; RMSEA = .06. Next, we examined the moderating role of country. Following Bollen’s (1989) suggestion, we first tested an unconstrained model where all path coefficients were allowed to vary between countries. Then we tested a fully constrained model where all path coefficients were fixed to be the same across the two countries. The unconstrained model had a significantly better fit than the fully constrained model, suggesting that at least some of the paths are not equivalent between the two countries. Then we tested a set of partially constrained models. In each model one path was set to be equal across the two countries. We then compared the fit of each constrained model with the baseline unconstrained model. In this way, we examined which specific paths were invariant across the two samples. Results of these analyses are shown in Table 3. The final structural paths are depicted in Figure 2. As shown in Table 3, the relations between the dummies contrasting the guilt-induction vignette with the other two vignettes and perceived controlling parenting differed between the two countries. Consistent with the MANOVA findings, Chinese adolescents perceived guilt-induction, relative to the generally controlling vignette, to be less controlling, whereas Belgian adolescents perceived both controlling vignettes to be equally controlling. Although both Belgian and Chinese adolescents perceived the guilt-induction vignette to be more controlling than the autonomy-supportive vignette, this effect was more pronounced in Belgian adolescents.

**Table 3:** Tests of Path Coefficient Equivalence between the Belgian and Chinese Samples.

*Model*	*SBSX^2^*	*df*	*CFI*	*SRMR*	*RMSEA*	*Model Comparisons*	
	
						*Comparison*	*Δ SBSX^2^*

Model 1: No constraints (baseline model)	715.62***	401	.93	.07	.05		
Model 2: Fully constrained model	815.45***	407	.91	.10	.06	vs. model 1	99.83***
Model 3: Fixed path from AS vs. Guilt-induction to PC	761.96***	402	.92	.07	.05	vs. model 1	46.34***
Model 4: Fixed path from Control vs. Guilt-induction to PC	720.33***	402	.93	.07	.05	vs. model 1	4.71*
Model 5: Fixed path from PC to Need Frustration	719.00***	402	.93	.07	.05	vs. model 1	3.38
Model 6: Fixed path from Needs to Compliance	743.93***	402	.92	.08	.05	vs. model 1	28.31***
Model 7: Fixed path from Needs to Defiance	716.04***	402	.93	.07	.05	vs. model 1	0.42
Model 8: Fixed path from Needs to Negotiation	740.53***	402	.93	.08	.05	vs. model 1	24.91***

*Note.* AS = Autonomy-Support; PC = Perceived Controlling style. *p < .05, **p < .01, ***p < .001.

Next we examined the association between perceived controlling parenting and need frustration. As shown in Table 3 and graphically displayed in Figure 2, this relation was equivalent across the two countries, with a perceived controlling style yielding a positive association with need frustration. These results support Hypothesis 2. Finally, we examined how need frustration was associated with the three coping reactions. As shown in Table 3, only the path between need frustration and oppositional defiance was equivalent across the two groups, with need frustration relating positively to defiance in both subsamples. Further, compulsive compliance was associated positively with need frustration in the Chinese sample, while it was unrelated to need frustration in the Belgian sample. Finally, as for negotiation, Chinese adolescents tended to use less negotiation in response to need frustration, whereas this relation was not significant for Belgian adolescents.

Finally, we also tested these differences between the two countries in a multivariate way, starting with the fully constrained model and setting free one more specific path in each subsequent model. This way we for instance could test whether the observed difference in the path from control vs. guilt-induction to perceived controlling parenting holds after releasing the other differences. These analyses completely confirmed the results in Table 3.

## Discussion

The present study aimed to extend our understanding of cultural similarities and differences in how adolescents perceive and cope with parental guilt-induction, relative to the use of generally controlling and autonomy-supportive parenting practices. To examine these processes, we made use of vignettes, which allowed us to operationalize parental practices in a standardized way. We chose to study guilt-induction in Chinese and Belgian adolescents because this practice was hypothesized to be especially prone to cross-cultural variability in its meaning and effect (e.g., Park & Kim, 2004; Rudy & Halgunseth, 2005). In general, we found that Chinese adolescents perceived parental guilt-induction, at least in the academic domain, as less controlling than Belgian adolescents. Yet, once the situations were perceived as controlling, adolescents from both countries suffered to a similar degree in terms of need frustration. Interestingly, they differed to some extent in the way how they coped with the experience of need frustration. Taken together, these findings shed new light on the underexplored micro-processes of how adolescents from different cultures cope with psychological need frustration.

### Cultural Differences in the Perception of Parental Guilt–Induction

As hypothesized, Belgian adolescents perceived the guilt-inducing vignette as equally controlling and need frustrating as the more generally controlling vignette. In addition, these two controlling vignettes both differed from the autonomy supportive vignette. This pattern of results suggests that for Belgian adolescents to experience feelings of parental pressure and need frustration, the use of guilt-induction suffices.

The pattern of findings among the Chinese adolescents was more nuanced, with the guilt-inducing vignette being perceived as moderately controlling and falling in between the generally controlling and autonomy-supportive situations. Specifically, Chinese adolescents perceived the guilt-inducing vignette to be less controlling and less need frustrating when compared to the generally controlling vignette, suggesting that parental guilt-induction, when used in isolation from other controlling practices, carries a less intrusive meaning. This is consistent with Rudy et al.’s (2014) finding that guilt-induction is associated less strongly with harshly controlling practices in people from collectivistic, relative to individualistic, societies. It is also consistent with recent factor-analytic evidence in a sample of Chinese-American mothers that guilt-induction is distinct from other psychologically controlling practices (shaming and love withdrawal) (Yu, Cheah, Hart, Sun, & Olsen, 2015). As highlighted by Park and Kim (2004), one reason could be that feeling guilty and indebted towards parents for their love and sacrifice is considered to be proper in Asian societies. Chinese adolescents may more easily internalize the moral obligations of reciprocal care and loyalty vis-à-vis their parents. As a result, when parents induce guilt in children by highlighting that they failed to reciprocate parents’ sacrifice and care, adolescents may accept this reasoning more easily and perceive it as less controlling. This may be especially the case for academic issues, as learning is viewed in China both as a moral endeavor to improve oneself and as a responsibility towards one’s parents (e.g., Li, 2005; Pomerantz & Qin, 2011). Promoting children’s learning is also viewed as a major responsibility for Chinese parents themselves, a task on which they spend considerable time (Chao, 1994). Given the moral importance of learning, parental investment in this domain may be interpreted less negatively in China and people may have more positive beliefs about guilt-induction. By inducing guilt, Chinese parents may believe that they highlight to their children the instrumental value of studying for school. Possibly, these parents hope that an initial sense of duty will give way to a volitional desire to study (Lens, Simons, & Dewitte, 2002).

However, it is noteworthy that although guilt-induction was perceived as less negatively than generally controlling behavior in Chinese adolescents, guilt-induction still was perceived as being more pressuring compared to the use of autonomy support. Chinese adolescents interpreted parental guilt-induction as more controlling than parental autonomy support and these heightened levels of perceived control, in turn, related to more need frustration with parents. This is consistent with Baumeister (1994)’s view that once people see guilt-induction as manipulative, they may respond with resentment and avoidance of the attachment figure.

An important question for future research is whether parental induction of guilt is related to adolescents’ personal proneness to guilt similarly across cultures. Because the induction of guilt by parents has a somewhat more mixed and benign connotation in collectivist cultures, it perhaps relates less strongly to painful personal feelings of guilt in those cultures compared to more individualistic cultures. Thus, future cross-cultural research could address the developmental sequence leading from parental guilt-induction, via children’s personal proneness to guilt, to children’s well-being and psychosocial adjustment. There is some evidence that, in spite of mean-level differences in personal guilt proneness between cultures (with Korean children for instance experiencing more guilt than US children), the intrapersonal experience of guilt is related similarly to developmental outcomes across cultures (Furukawa, Tangney, & Higashibara, 2012). Fontaine et al. (2006) also found that the relations between the intrapersonal experience of guilt and various other subjective experiences were remarkably similar among samples from Belgium, Peru, and Hungary. Given the limited number of studies on this issue, however, clearly more research is needed.

### Cultural Similarities in Perceived Psychological Need Frustration

In spite of the cultural differences in the way parental guilt-induction is perceived, some of the subsequent processes elicited by perceived controlling parenting were similar across both cultures. Specifically, the association between perceived controlling parenting and psychological need frustration was equivalent in Belgium and China. This finding is consistent with the assumption in SDT about the negative effects of *perceived* parental pressure across cultures (Grolnick et al., 1997; Soenens et al., 2015). The current findings are consistent with previous studies documenting negative effects of perceived controlling parenting across cultures (Pomerantz & Wang, 2009; Soenens et al., 2012). The present study extends this work by showing that, although Chinese adolescents perceived guilt-induction to be less controlling than Belgian adolescents, once participants perceived the situation as controlling, regardless of their cultural background, they did suffer from it in terms of need frustration.

### Cultural Similarities and Differences in Coping with Perceived Need Frustration

Although the experience of need frustration has been found to yield various costs (Vansteenkiste & Ryan, 2013), few studies have examined how people actively cope with such experiences of need frustration (Skinner & Edge, 2002). Therefore, we investigated three different coping reactions. Chinese adolescents displayed an intriguing mix of responses. Specifically, they reported both being more inclined to comply and to defy the request to study more. Further, they also reported being less likely to start negotiating with parents. This pattern of responses was in certain ways similar but in others ways dissimilar from how the Belgian adolescents reacted. Specifically, also the Belgian adolescents reported they would engage in oppositional defiance. Yet, differently from the Chinese adolescents, they would not comply compulsively with the parents and would not feel inhibited to negotiate.

As for the specific coping responses, in the Chinese sample, but not in the Belgian sample, negotiation was related negatively to perceived need frustration. This finding indicates that, while Chinese adolescents are unlikely to negotiate with their parents when experiencing need frustration, need frustration does not preclude negotiation among Belgian adolescents. Possibly, Belgian adolescents grow up within a relatively democratic family culture which is more prevalent in European – Western society (Oyserman et al., 2002), and such a democratic atmosphere offers relatively more space to negotiate. If we interpret this pattern in a reversed way, it indicates that Chinese adolescents only tend to negotiate if they perceived more satisfaction in the situation. Probably in such a case, they feel more psychologically secure to negotiate and to assert their preference and opinions. Future research could address the specific reasons why Chinese adolescents tend to negotiate less. Factors that could be relevant are the more hierarchical nature of parent-child relationships in China and adolescents’ feeling that their parents sacrifice a lot to support their academic success.

Different from our hypothesis, we found that Chinese adolescents tended to adopt similar levels of oppositional defiance compared to Belgian adolescents when they perceived need frustration in the vignettes. This is surprising because Chinese adolescents are said to be more oriented towards interdependence and obligation vis-à-vis parents (Pomerantz & Qin, 2011). Previous studies have documented that perceived controlling parenting relates to oppositional defiance in Western samples (Van Petegem et al., 2015). The present study added to these findings by showing that Eastern Asian adolescents also tended to rebel against controlling parenting. Although Chinese and Belgian adolescents appear to respond to need frustration with similar levels of defiance, it might still be the case that the manifestation of defiance differs. For instance, compared to Belgian adolescents Chinese adolescents may engage in relatively more covert defiant behaviors that violate social norms in less visible ways. In this regard, it is interesting to note that the correlation between oppositional defiance and compulsive compliance was not significant in the Chinese sample, indicating that it is possible for a Chinese adolescent to adopt both defiance and compulsive compliance in the face of pressuring experiences. Although perhaps contradictory at first sight, these findings indeed suggest that it is possible for Chinese participants to be compliant to parents on the surface, yet to seek for covert ways to rebel against parental authority at the same time. If this is true, Chinese adolescents would seek more insidious and advanced ways of being defiant (e.g., being defiant behind the parents’ back). It is also possible that Chinese participants tend to have both reactions, albeit not necessarily simultaneously. They could be compliant on one day and rebel the next day. Another explanation could be that we operationalized the coping responses as the hypothesized intention to adopt the reaction in the vignette rather than in terms of actual coping behavior. Thus, it is possible that Chinese adolescents have the intention to rebel yet do not actually put this intention into practice. Future research is needed to directly address these possibilities.

When Chinese adolescents perceived more need frustration, they tended to comply more. This is consistent with the finding that they tended to negotiate less when experiencing need frustration. In contrast, for Belgian adolescents, perceived need frustration with parents was not related to compliance, suggesting that Belgian adolescents did not systematically adopt compulsive compliance as a coping strategy for need frustration. Although compliance may seem like a culturally proper reaction in China because it involves filial piety and is aimed at maintaining harmony with parents (e.g., Rothbaum et al., 2000), it is interesting to see that compulsive compliance actually was closely and positively linked with perceived need frustration in China. Our data do not allow us to make firm conclusions about the maladaptive or adaptive nature of compulsive compliance as a coping response for Chinese adolescents because we did not examine outcomes of this coping response. Yet, its association with psychological need frustration seems to point to its maladaptive nature. Future longitudinal research is needed to further investigate the functional role of this coping strategy.

It is important to note that we focused on compulsive compliance, which is defined and operationalized as rigid obedience with pressure (Grusec & Kuczynski, 1997; Skinner & Edge, 2002). It is also possible that some adolescents comply because they fully agree with parents’ requests and feel willing to do follow the requests. Such a “willing submission” has been referred to as accommodation, a constructive coping style involving genuine acceptance and cooperation (Kochanska & Aksan, 1995; Skinner & Edge, 2002). It would be interesting for future research to investigate both types of compliance simultaneously.

### Limitations and Future Research Directions

A number of limitations warrant interpreting the current findings with caution and suggest directions for future research. One limitation is that we relied on participants’ self-reports as a single source of information about all study variables. This exclusive reliance on self-report may have artificially inflated some of the observed relations. Yet, because our study focused on adolescents’ intrapsychic perception of parental behavior and their intention to cope with need frustration, we believe that self-reports are more relevant than other informants’ reports.

Second, we did not assess the cultural values of collectivism or filial piety presumed to underlie the cultural difference in adolescents’ interpretation of parental guilt-induction, nor did we assess adolescents’ parenting history. This limitation precludes a further explanation of why Belgian and Chinese adolescents differ in their perception and coping towards parental control. For instance, adolescents with a relatively autonomy-supportive history of parenting might tend to use more negotiation when faced with unfair and need frustration experiences. The possible role of individuals’ history of parenting definitely warrants future research.

Third, the two cultural samples were recruited with different procedures and this might have created potential variance that is not culturally relevant. Furthermore, we did not include measures of socio-economic status (SES). Although previous research with similar samples and recruitment procedures displayed only minor differences in SES (e.g. parental educational level) between Chinese and Belgian samples (Wuyts, Chen, Vansteenkiste, & Soenens, 2015), we cannot exclude the possibility that differences in SES also contributed to differences between the two cultural groups in terms of the study variables. Fourth, the scenarios used in the current study focused only on parental involvement in adolescents’ academic performance. Yet, we need to be cautious in generalizing the results to other life domains. Future research can investigate adolescents’ perception and coping across different social domains to see whether there are interactions between domain and culture (Smetana, 2000).

Finally, the current investigation of adolescents’ coping with perceived need frustration in scenarios was somewhat preliminary. We only examined adolescents’ intention to use various coping responses in hypothesized situations. It is unclear what these coping responses really mean to them, what their developmental outcomes are, and whether they are actually enacted in behavior. Future research can further explore the meaning of defiance, compulsive compliance, and negotiation to adolescents in different cultures. Further, it would be interesting to include a more constructive type of compliance reflecting the construct of accommodation (Skinner & Edge, 2002). Investigating these coping responses is important because it may provide more insight into the reason why some adolescents suffer more than others from controlling parenting and why some might even be capable of displaying an adaptive coping response. Longitudinal research would be ideally suited to examine adolescents’ actual coping behavior in daily life and to further highlight adolescents’ active role in strengthening or breaking the recursive loop of controlling parental behaviors and personal maladjustment.

## Conclusion

Conciliating cultural-specific and universal-process perspectives on parenting, the present study suggested that cultural differences in the dynamics of parenting mainly lie (a) in the different degree to which adolescents perceive a potentially controlling parental practice such as guilt-induction as being actually controlling and (b) in the different ways in which they cope with the experiences following from perceived controlling parental behavior. Notwithstanding these cross-cultural differences, perceived controlling parenting also seemed to have a common function as it was related to psychological need frustration in similar ways across the two cultures.

The findings from this study have a number of implications for counseling with parents and adolescents and in particular for immigrant families. First, parents could be made aware that, in general, guilt-induction is perceived as more “pressuring” than autonomy support. More importantly, once adolescents perceive this sense of “pressure” or “manipulation” to make them comply with parents’ expectations, they suffer from it despite of the culture of origin. For healthy parent-child relationships and adolescents’ psychological well-being, it is advised not to use guilt-induction, but instead to engage in more autonomy-supportive practices. Second, in schools or in clinical counseling with adolescents from culturally diverse backgrounds, counselors need to realize that these adolescents could have different interpretations of parents’ behavior depending on their cultural background. Also, counselors could try and take into account the fact that culture plays a role in how children cope with controlling parenting. Yet, underlying the variations in interpretation of and coping with parental behavior, the psychological costs associated with perceived controlling parenting are essential and universal.

## Competing Interests

The authors declare that they have no competing interests.
